# Dual Sacrificial Molding: Fabricating 3D Microchannels with Overhang and Helical Features

**DOI:** 10.3390/mi9100523

**Published:** 2018-10-16

**Authors:** Wei Huang Goh, Michinao Hashimoto

**Affiliations:** 1 Engineering Product Development, Singapore University of Technology & Design, Singapore 487372, Singapore; weihuang_goh@mymail.sutd.edu.sg; 2 Digital Manufacturing and Design (DManD) Centre, Singapore University of Technology and Design, Singapore 487372, Singapore

**Keywords:** microfluidics, sacrificial molding, 3D printing, 3D microchannel, surface roughness

## Abstract

Fused deposition modeling (FDM) has become an indispensable tool for 3D printing of molds used for sacrificial molding to fabricate microfluidic devices. The freedom of design of a mold is, however, restricted to the capabilities of the 3D printer and associated materials. Although FDM has been used to create a sacrificial mold made with polyvinyl alcohol (PVA) to produce 3D microchannels, microchannels with free-hanging geometries are still difficult to achieve. Herein, dual sacrificial molding was devised to fabricate microchannels with overhang or helical features in PDMS using two complementary materials. The method uses an FDM 3D printer equipped with two extruders and filaments made of high- impact polystyrene (HIPS) and PVA. HIPS was initially removed in limonene to reveal the PVA mold harboring the design of microchannels. The PVA mold was embedded in PDMS and subsequently removed in water to create microchannels with 3D geometries such as dual helices and multilayer pyramidal networks. The complementary pairing of the HIPS and PVA filaments during printing facilitated the support of suspended features of the PVA mold. The PVA mold was robust and retained the original design after the exposure to limonene. The resilience of the technique demonstrated here allows us to create microchannels with geometries not attainable with sacrificial molding with a mold printed with a single material.

## 1. Introduction

This paper describes a method to fabricate microchannels with three-dimensional (3D) overhang features by performing sacrificial molding twice. We termed this process dual sacrificial molding. Dual sacrificial molding is a process whereby a mold consisting of two printable materials were sacrificed sequentially to impart the desired pattern embedded in another matrix. The developed method allowed fabricating microchannels with features that consisted of protruding or fully suspended structures, such as 3D overhangs and helices in the desired matrix. To date, the complexity of microchannels fabricated by sacrificial molding is limited by the geometry of the removable mold. The method we report allows overcoming the restriction of the attainable geometry of the sacrificial mold so that 3D geometry of microchannels can be readily fabricated. 

Conventional techniques for the fabrication of microfluidic devices use replica molding to fabricate two-dimensional (2D) geometry of microchannels complementary to molds patterned by photolithography [1]. By replica molding and soft lithography [2], fabrication of 3D microfluidic devices would require precise alignment and laborious stacking of multiple layers of the matrix consisting of complimentary parts contributing to the final assembly [3,4]. Multiple methods such as etching, molding and sintering may be combined to produce 3D complex structures, albeit with rigorous alignment [5]. Recently, 3D printing, one of the most widely used techniques in additive manufacturing, was demonstrated to fabricate microfluidic devices harboring 3D geometry of microchannels. Examples of 3D printing capable of producing an entire microfluidic device include stereolithography [6], polymer jet printing [7], and fused deposition modeling (FDM) [8,9]. Alternatively, microfluidic devices can be fabricated by evacuating microscale mold embedded in a matrix of interest. This process is known as sacrificial molding [10,11,12]. Using this approach, sugar-based substrate [12], acrylonitrile butadienestyrene [13], organic or aqueous-based solutions [14,15], and eutectic metal [16] have been used as sacrificial materials to create microchannels with controlled dimension. FDM 3D printing has been used to pattern complex, intricate 3D features of sacrificial molds such as staggered herringbones to fabricate micromixers embedded in the microchannels [10].

Despite all successful demonstrations by sacrificial molding to produce microchannels in a matrix, the fundamental designs attained were still limited to the geometry conferred by the sacrificial mold. Conversely, the printed geometry of the sacrificial mold may be constrained by the capability of the instrument handling the production of the mold. Fabrication of sacrificial materials has been demonstrated using direct ink writing (DIW) and FDM 3D printers. With DIW 3D printers, liquid sacrificial materials are directly patterned in other matrices, and it is not possible to obtain a robust 3D structure of sacrificial molds. The basic principle of FDM 3D printing involves fabricating an object in a layer-by-layer manner in the vertical dimension until the final structure is achieved. Specifically, a subsequent layer of an extruded material is printed on the support by the exterior surface of the previously printed structure. However, a notable challenge arises when one fabricates a design consisting of 3D geometries suspended in space. In the interests of fabricating microchannels by sacrificial molding, 3D features of microchannels such as overhangs or a helix would be difficult to be made. The suspended features would require a prior layer of sacrificial material beneath the area where the features would be printed. Thereafter, the prior layer is removed to reveal the overhang features.

To date, fabricating microchannels with overhang and helical features by sacrificial molding is still challenging and requires the use of auxiliary tools. For example, a mold made by dispensing fugitive ink onto a rotating template was used to produce helical structures that would become helical microchannels after sacrificial molding in epoxy matrix [17]. Similarly, a mold resembling a helical structure can be printed by polymer jetting and then manually pulled out of the castable matrix [18]. Alternatively, overhang structures can be created post-fabrication by heating and manipulating material to the desired shape by hand [13]. Despite those demonstrations, fabrication of 3D microchannels by sacrificial molding has been limited by the lack of flexible methods to create arbitrary shape of 3D molds using robust sacrificial materials.

To overcome this limitation, herein we developed a method termed dual sacrificial molding that permitted to fabricate 3D microchannels using complex geometry of sacrificial molds. Dual sacrificial molding relies on the complementary pairing of two sacrificial materials and respective solvents used to remove them. We used HIPS as the first sacrificial material (Sacrificial Material 1 or SM1) that was removed in limonene (a type of terpenes). PVA was used as the second sacrificial material (Sacrificial Material 2, or SM2) that was removed using water. SM1 served as the support material for SM2 during the FDM printing session, while SM2 contained the design of microchannels to be fabricated. We demonstrated the proof-of-principle that microchannels were fabricated in the matrix of polydimethylsiloxane (PDMS) using the proposed method and materials. The present work harnessed the potential of using ubiquitously available FDM printing and typical filament as sacrificial materials to fabricate complex 3D microchannels in a range of matrices [10]. Specifically, the ability to create sacrificial molds with complex 3D features with PVA—which is soluble in water and capable of fabricating microchannels in matrices such as cell-laden hydrogels—has been limited to date. Our method provided a facile way to fabricate PVA sacrificial molds with complex 3D geometries with a simple FDM 3D printer. This capability would be potentially useful in fabricating 3D microchannels in various matrices, including biofabrication of 3D vascularized structures in hydrogels.

## 2. Experimental Design

The objective of the current work is to apply sacrificial molding to fabricate microchannels with an overhang and 3D suspended features such as helices. The suspended nature of these structures posed challenges to print entirely using a single material. As we reported previously, sacrificial molding with a 3D printed mold made of PVA is an efficient and benign method to create 3D microchannels within a multitude of matrices [10]. Thus, the challenge is to extend sacrificial molding with PVA molds complemented by a secondary material that aids in the fabrication of the molds with suspended geometry.

The principle of fabricating dual sacrificial molds for microchannels with overhangs or helical geometry hinges on the appropriate pairing of materials used for printing by FDM 3D printer and the solvents used to dissolve the mold. The two materials selected should exhibit adherence between them while they are printed and should be resistant to solvents used to remove the other materials. As the geometry of overhangs and helixes lay suspended in air, the use of a support material was necessary to prevent the overhang structure from deforming or collapsing while the material is being extruded by the hot nozzle. More importantly, preliminary work suggested molten PVA adhered well on HIPS, and molten HIPS adhered well on PVA. We selected PVA as the main sacrificial material to ensure that microchannels can be created in various matrices including hydrogels. The use of PVA ensured the compatibility of the method for fabrication of microfluidic devices suitable for a plethora of applications in chemistry and biology.

## 3. Materials and Methods

### 3.1. Design and Fabrication of Dual 3D Sacrificial Mold

Microchannels with serpentine, dual helix and multilayer pyramidal network were designed using 3D computer-aided design (CAD) software (AutoCAD 2015, Autodesk, Inc., San Rafael, CA, USA). Individual CAD designs were exported to a file format known as the standard triangulation language (STL), which was subsequently converted into individual layers by Makerbot Desktop software (MakerBot Industries, New York, NY, USA) to form G-codes. Prior to generating a file containing G-codes, we utilized the dual extrusion feature in the software by activating “support function” in the slicer software. By activating *support function*, the left extruder extruded HIPS and the right extruder extruded PVA. Thereafter, the G-codes were generated and sent to the ROKIT 3dison multiprinter (ROKIT, Seoul, South Korea) to fabricate a mold containing HIPS and PVA.

The bed of the printer was leveled by manually adjusting the screws at four corners of the print bed. A paper with a thickness of 100 μm was used as a spacer between the nozzle outlet and the bed to calibrate first layer height of 100 μm. The first layer height was critical as it ensured that the extruded filament has sufficient adhesion onto the print bed. PVA filament (SAINSMART, Lenexa, KS, USA) was trimmed into strips of around 100 cm and baked overnight in an oven at 60 °C before printing. The diameter of the nozzle on both extruders was 200 μm, and the temperature of the right nozzle was programmed to 182 °C and the left nozzle was programmed to 230 °C. The object infill parameter was set to 100% to obtain a solid print. The layer height was set at 100 μm. The temperature of the print bed was programmed to 85 °C to prevent warping of HIPS from the print bed. The door to the print chamber was closed until the completion of printing. The dual sacrificial mold was removed from the print bed immediately after the printing was complete.

### 3.2. Fabrication of 3D Microchannels in PDMS

The dual sacrificial mold was submerged in a bath containing limonene (Sigma-Aldrich, Singapore). The solvent was stirred gently at room temperature to dissolve HIPS and reveal the structure made of PVA. Next, the mold was removed from the bath containing limonene and rinsed with fresh volumes of limonene to remove any HIPS residues. The mold consisting of PVA only was rinsed with fresh volume of ethanol (Sigma-Aldrich) and allowed to dry under a gentle stream of compressed air. The PVA mold was visually inspected under a Leica stereoscope (Leica Microsystems GmBH, Wetzlar, Germany) coupled with a Basler AG camera (Ahrensburg, Germany) to detect any HIPS residue. The PVA mold was then stored in an airtight container until use to prevent premature degradation due to moisture present in the ambient air.

PDMS was prepared by mixing PDMS prepolymer and the curing agent (Sylgard 184, Dow Corning, Midland, MI, USA) in a ratio of 10:1 by weight. A base layer was cast and cured before use. PDMS was then poured gently until the PVA mold was completely embedded. The PDMS and embedded mold were degassed under vacuum for 30 min and then allowed to cure at 60 °C for 2 h. After the PDMS has cured, inlets and outlets were created on the PDMS devices by coring a hole with a 1.5 mm outer diameter biopsy punch (Ellis Instruments, Madison, NJ, USA). The PVA mold, which was embedded in the PDMS matrix, was submerged and subjected to ultrasonic treatment in a water bath (Symphony Ultrasonic bath, VWR, Padnor, PA, USA) to remove the PVA mold at room temperature. The PDMS devices were ready for use after the PVA mold was completely removed.

### 3.3. Characterization of 3D Microchannels

Characterization of optical images of the microchannel was performed on a Leica stereoscope (Leica Microsystems GmBH) coupled with a Basler AG camera. A calibration of image was performed using a standard ruler (MU500, Amscope, Irvine, CA, USA) and used as a calibration guide in Fiji software [19]. A cross section of the overhang segment of the microchannels created in PDMS after removal of the mold was evaluated by taking measurements at the longest dimension of width (horizontal dimension). Data were analyzed in Microsoft Excel (Microsoft Corporation, Seattle, WA, USA) and presented as mean ± SD.

### 3.4. Surface Roughness and 3D Profiling of PDMS Microchannel with Overhang Features

Surface profiling of the microchannel was performed on the HIROX KH-8700 Next Generation 3D digital microscope (Hirox, Tokyo, Japan). Roughness measurement was assessed using the three parameters (R_a_, Arithmetical average roughness; R_z_, Maximum height; and R_zjis_, 10-point mean roughness available in the HIROX software. The PDMS microchannel was sliced and the surface of the microchannel of interest was placed on a fixing tape. The surfaces of the microchannel were assessed in the top-down perspective. The images were quantified with the OL350(II) objective lens (Hirox, Tokyo, Japan) with a magnification of 350×. Prior to 3D image analysis, the lowest and highest focal points were established, and a 3D model was acquired by setting the integrated stepping motor to scan in steps of 2 µm. Thereafter, automatic scanning was initiated and the images were rendered in a 3D model for analysis. The 3D model was displayed as pseudo color to highlight the surface profile solely for viewing purposes. A 3D profile line was drawn on the 3D image (horizontal dimension) to analyze surface roughness. Data were analyzed in Microsoft Excel (Microsoft Corporation, Seattle, WA, USA) and presented as mean ± SD.

### 3.5. Perfusion of Fluids to Reveal Microchannels with Overhang and Helical Geometry

The red dye solution was prepared at 10% (*v*/*v*) by mixing red dye (Star Brand, Kuala Lumpur, Malaysia) with glycerol (Sigma-Aldrich). For visualization of fabricated microchannels, a solution of red dye was manually perfused using a syringe that was connected to sterile needles (Terumo, Tokyo, Japan) and PTFE tubing (Cole-Parmer Instrument Co., Chicago, IL, USA) to fill the PDMS devices.

## 4. Results and Discussion

### 4.1. Fabrication of Microchannels by Dual Sacrificial Molding Method in Castable Matrix

**Figure 1:**
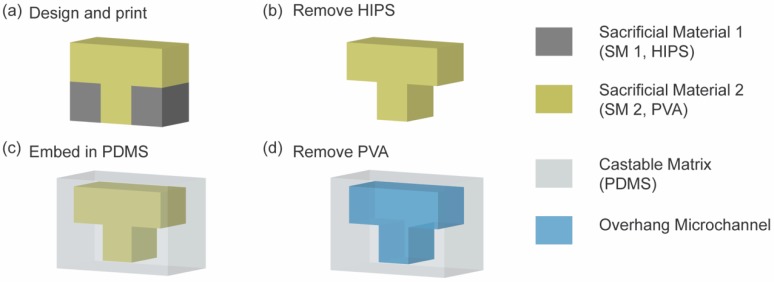
Schematic illustrations of dual sacrificial molding of HIPS and PVA to fabricate microchannels with an overhang or helical features in a castable matrix. The process of dual sacrificial molding can be divided into two major phases: (1) removal of Sacrificial Material 1 (HIPS, grey) (**a**,**b**), and (2) removal of Sacrificial Material 2 (PVA, pale yellow) (**c**,**d**). (**a**) An overhang pattern was printed by dual extrusion of HIPS and PVA. HIPS was used as a support material during FDM printing; (**b**) HIPS was removed in limonene, and the PVA sacrificial mold was obtained; (**c**) The PVA sacrificial mold, containing the overhangs, was embedded in PDMS; (**d**) PVA was removed in an ultrasonic water bath, and microchannels with overhangs were obtained.

The concept of dual sacrificial molding to fabricate microchannels with overhang is illustrated in Figure 1. We termed the process of fabrication as dual sacrificial molding to highlight two different stages to remove the printed materials: (1) sacrificing HIPS from the 3D printed structure to reveal the PVA mold; and (2) sacrificing the PVA mold after it was embedded in PDMS. Microchannels with desired 3D features were designed by a computer-aided design (CAD) software and printed by a FDM 3D printer. Two different sacrificial materials were used to print dual sacrificial mold consisting of the microchannel with overhang geometry. SM1 (HIPS) was printed in the surrounding space to support SM2 (PVA) (Figure 1a). Then, the 3D printed mold was submerged in a bath containing limonene to dissolve HIPS completely to reveal the PVA sacrificial mold. Thereafter, the PVA sacrificial mold was carefully removed from the limonene bath and inspected under a microscope to ensure that the mold was devoid of the residue of HIPS. It was critical to inspect the sacrificial mold visually to confirm that HIPS was completely dissolved from the surface of PVA mold. Subsequently, the PVA mold was allowed to dry overnight in a dry box. After this step, the PVA sacrificial mold was free from HIPS (Figure 1b). For the second step of fabrication, the PVA mold was carefully placed on a layer of cured PDMS and then embedded in uncured PDMS mixture and left to cure for 2 h (Figure 1c). Thereafter, an inlet and outlet were created to expose the PVA sacrificial mold to water in an ultrasonic bath. Finally, the PVA sacrificial mold was removed, and the void spaces created in PDMS served as microchannels (Figure 1d).

### 4.2. 3D Printing of Sacrificial Mold Patterned with Overhang Features

**Figure 2:**
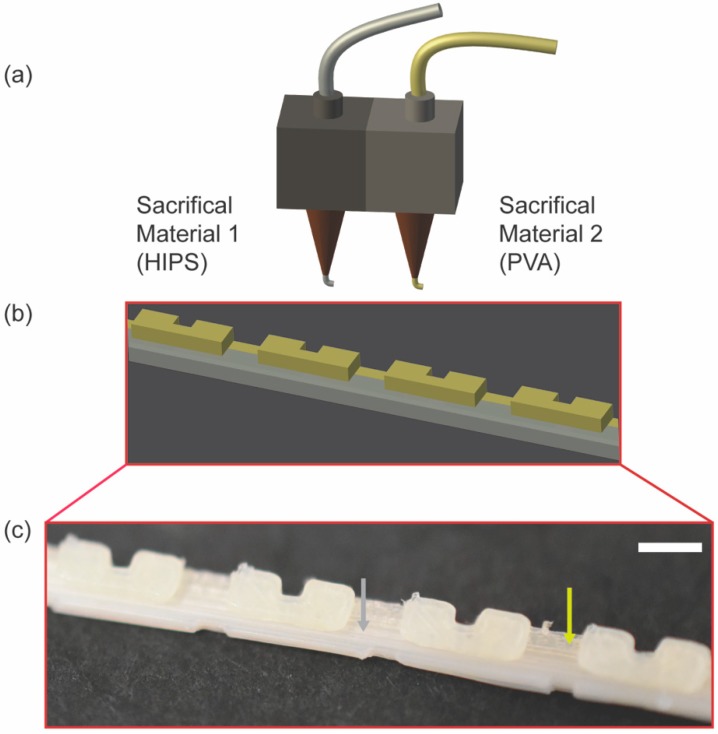
Schematics and optical images showing fabrication of dual sacrificial mold by dual extrusion printing using a FDM 3D printer. (**a**) Schematic of dual extruder installed on the 3D FDM printer dispensing HIPS as Sacrificial Material 1 from the left extruder and PVA as Sacrificial Material 2 from the right extruder; (**b**) A 3D model of a sacrificial mold with a serpentine pattern printed by dual extrusion of HIPS and PVA. The support structure shaded in grey was printed with HIPS and the channel structure shaded with yellow was printed with PVA; (**c**) Optical image of the printed model depicting deposition of HIPS (indicated with a grey arrow) and PVA (indicated with a yellow arrow). Scale bar = 2.0 mm.

To obtain a prototype of PVA sacrificial mold patterned with overhang features, a FDM 3D printer with two hot-end nozzles was required. Generally, the dual sacrificial mold is fabricated by two extruders extruding two different materials (Figure 2a). This concept is illustrated by the schematic showing the site of deposition of SM1 and SM2 on a dual sacrificial mold (Figure 2b). It is imperative that the materials selected for dual extrusion by a FDM 3D printer complement each other in terms of the layer to layer adhesion between the two distinct materials of choice. In particular, HIPS must adhere to the previously extruded layer of PVA, and conversely, PVA must adhere to the previously extruded layer of HIPS for dual extrusion printing to be successful. Experimentally, we observed good adhesion between the two materials, suggested by the corresponding image of the prototype of the mold consisting of HIPS and PVA (Figure 2c).

### 4.3. Characterization of Overhang Feature of Serpentine Microchannel

**Figure 3:**
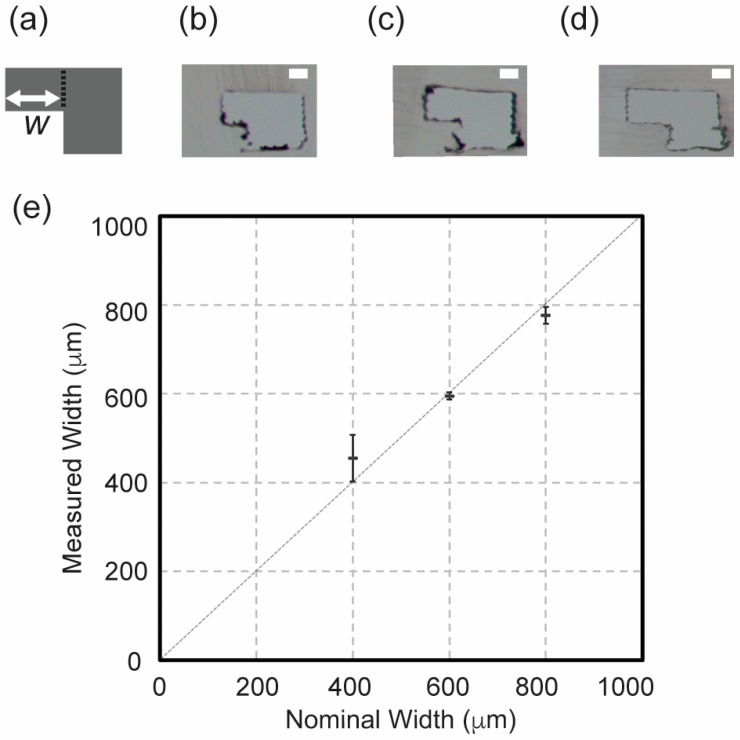
Characterization of the width of the overhangs in the serpentine microchannel fabricated by dual extrusion of HIPS and PVA. (**a**) Schematic showing a cross section of the overhang feature that was studied. The width of the overhang of the microchannel (*w*) was characterized. Representative images of cross sections of overhang features fabricated in PDMS with the overhang (**b**) 400 µm, (**c**) 600 µm, and (**d**) 800 µm. (**e**) Characterization of the variation of the width of the fabricated overhangs with respect to the nominal width designed by CAD. The data are mean ± SD (*n* = 10). Scale bar = 400 µm.

In order to confirm that with the overhang features were attainable with dual sacrificial molding, we fabricated sacrificial molds with the design of serpentine microchannels and varied the nominal width of the overhangs printed in PVA in 400, 600, and 800 µm with HIPS as a support material (Figure 3a). After the removal of HIPS, the PVA mold was embedded in PDMS and removed in water. Although the visual inspection revealed that the PVA mold was removed from PDMS matrix, it is conceivable that residual PVA molecules remained on the surface of PDMS due to adsorption [20]. The microscopic images depict the cross section of the microchannels with overhangs in PDMS after dual sacrificial molding. The morphology of the microchannels with overhang is shown (Figure 3b–d). We characterized the nominal dimension of the width of the channel with overhangs and the measured width of the channel in PDMS (Figure 3e). The measurement of the dimensions of the features was done by the image analysis using a calibration ruler. An image of the calibration ruler was taken under the settings used to capture all images of samples analyzed in Fiji software. Under the experimental condition, the scale was set to 5.5 μm per pixel, determining the accuracy of the image analysis by the microscope discussed in this paper. The mean widths of the overhangs were obtained as 455 µm for 400 µm (standard deviation (SD) = 52 µm), 595 µm for 600 µm (SD = 8 µm) and 777 µm for 800 µm (SD = 19 µm) for three widths of the overhangs designed in CAD. The standard deviations in widths we obtained by dual sacrificial molding are comparable to 41 µm we reported previously using PVA molds [10]. Although we showcased dimension ranging from 400 µm to 800 µm in this paper, previous study had demonstrated that it is plausible to create PVA sacrificial mold with a dimension of 200 µm in width [10]. Conversely, dimensions above 800 µm could be attained, albeit restricted by the size of the printing platform of the FDM 3D printer.

Since PVA is poorly soluble in most organic solvent [21], we postulated that limonene can be removed without damaging the PVA mold. The design of the PVA sacrificial mold defined the final outcome of the microchannel created in the PDMS matrix. The measurement of the width of the overhangs suggested that the dimension of PVA was not affected by the exposure to limonene. We therefore concluded that dual sacrificial molding had no negative impact on the designs of microchannels created in the PDMS matrix for the current choice of the materials.

### 4.4. Surface Roughness of Microchannel Fabricated by Dual Sacrificial Molding

The microchannels fabricated by dual sacrificial molding had grooves along the vertical wall due to the layer-by-layer deposition of the filaments during FDM 3D printing of the mold. This observation was consistent with the previous demonstration of sacrificial molding to fabricate microchannels [10]. In particular, the pattern on the surface of FDM printed mold is determined by the style of deposition of the filament during printing. In this work, we selected the linear infill pattern and a 100% infill density to produce a solid mold. As a result, the filament was deposited in straight lines with the width of 200 µm, alternating between the X-direction and the Y-direction. On the other hand, the patterns originating from the layer-by-layer stacking of the filaments was retained in the Z- direction in the dimension of 100 µm; this dimension was selected as the layer height for the 3D printing. To this end, the resultant design of the microchannels fabricated by sacrificial molding of the FDM 3D-printed mold was ultimately determined by the mechanism and resolution of the FDM printing.

**Figure 4:**
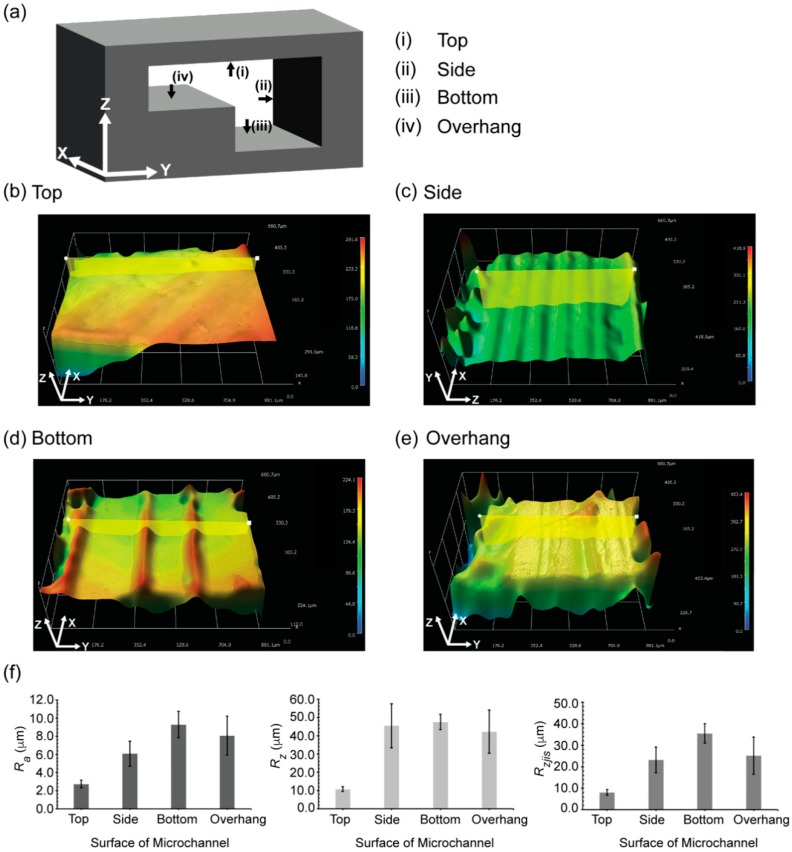
3D profile of the surfaces of the microchannel fabricated by dual sacrificial molding of HIPS and PVA mold. (**a**) Schematic of the cross section of the microchannel with an overhang feature. Four surfaces of the microchannel were analyzed to study surface roughness: (i) the top surface of the microchannel, (ii) the side wall of the microchannel, (iii) the bottom surface of the microchannel and, (iv) the bottom surface of the overhang feature; (**b**) 3D profile of the top surface of the microchannel; (**c**) 3D profile of the side wall of the microchannel; (**d**) 3D profile of the bottom surface of the microchannel; (**e**) 3D profile of the bottom surface of the overhang feature of the microchannel; (**f**) Plots showing three parameters to characterize surface roughness: (left) R_a_ values representing the mean roughness of the surface, (middle) R_z_ values representing maximum height of roughness of the surface and (right) R_zjis_ values representing 10-point mean roughness of the surface profiled by HIROX software. The data are mean ± SD (*n* = 6).

We profiled the four surfaces of the fabricated microchannels: (1) the top surface (originating from the last layer of the PVA filament, Figure 4a(i)); (2) the sidewall (originating from the stacking of the PVA filament, Figure 4a(ii)); (3) the bottom surface (originating from the first layer of the PVA filament deposited on the printing bed, Figure 4a(iii)); and (4) the overhang surface (originating from the interface between the HIPS layer and the PVA layer, Figure 4a(iv)). The morphology of the exterior of the FDM printed mold was responsible for the morphology of the inner surface of the microchannel.

Based on the 3D profile of the surface topology, we observed linear grooves at the bottom of the microchannel (Figure 4d) and at the bottom of the overhang feature (Figure 4e). This observation was consistent with the infill patterns programmed for the print. Repeating groove-like patterns resembling the layer-by-layer stacking of the filament were also observed on the side wall of the microchannel (Figure 4c). We observed groove-like patterns in the diagonal orientation on the top surface of the microchannel (Figure 4b), which reflected the printing pattern at the top surface of the sacrificial mold. Due to the mechanism of the FDM printing, the inner surfaces of the microchannels were uneven. This observation is consistent with the previous study investigating the surface roughness of the microfluidic devices directly printed by FDM [7]. To avoid these uneven surfaces, post-processing methods may be harnessed to reduce the surface roughness of FDM 3D printed parts [22]. In the current study, we assessed the three parameters associated with surface roughness (R_a_, R_z_ and R_zjis_). The mean R_a_ values were 2.7 µm (SD = 0.7 µm) for the top surface of the microchannel, 6.1 µm (SD = 1.4 µm) for the side wall of the microchannel, 9.3 µm (SD = 1.5 µm) for the bottom surface of microchannel, and 8.1 µm (SD = 2.1 µm) for the bottom surface of overhang feature. The R_a_ values of the surface of the microchannels fabricated in this study were comparable to the R_a_ value of 12.6 µm measured for the microfluidic device directly printed by FDM 3D printer [7].

Due to the presence of the groove-like features on the surface of the microchannel, we investigated the R_z_ values to assess the maximum roughness observed on the surface. The mean values of R_z_ were 10.8 µm (SD = 1.3 µm) for the top surface of the microchannel, 45.5 µm (SD = 12.0 µm) for the side wall of the microchannel, 47.6 µm (SD = 4.2 µm) for the bottom surface of the microchannel and 42.3 µm (SD = 11.7 µm) for the bottom surface of the overhang feature. Finally, we characterized the R_zjis_ values that provided the mean roughness of 10 randomized spots on the surface profiled. The R_zjis_ values was 8.1 µm (SD = 1.3 µm) for the top surface of the microchannel, 23.2 µm (SD = 6.0 µm) for the side wall of the microchannel, 35.5 µm (SD = 4.4 µm) for the bottom surface of microchannel and 25.2 µm (SD = 8.6 µm) for the bottom surface of overhang feature. As the surface roughness of the bottom surface of the overhang feature and the bottom surface of the microchannel were similar, the use of HIPS as a support material for the overhang feature did not alter the surface roughness of the resulting PVA mold. Interestingly, the surface roughness of the top surface of the microchannel appeared to be the least among the four surfaces that were characterized. The top surface of the microchannel was complementary to the open surface of the last layer of the PVA filament (in contrast to the PVA filaments deposited on other surfaces such as the printing bed and the existing HIPS layer). This difference may explain the low surface roughness of the top of the microchannel. 

We characterized the roughness of the surfaces parallel to those defined by the axes of the coordinates (e.g., XY and XZ planes). In these cases, the surface roughness of the resulting microchannels can be governed by either the printing profile or the stacking of the filaments. In general, the surface roughness of the microchannels with arbitrary geometries can be higher than that of the simple planes. For example, the mold of the helical microchannel requires printing of PVA on HIPS in a diagonal direction in one layer; the printing of PVA continues with some offsets in the subsequent layers. In such case, the surface roughness would be attributed to both the printing profile and the stacking of the filaments between layers, and the resulting values should be higher than those of the planes defined by the axes.

### 4.5. Examples of Microchannels with 3D Overhangs and Helices

**Figure 5:**
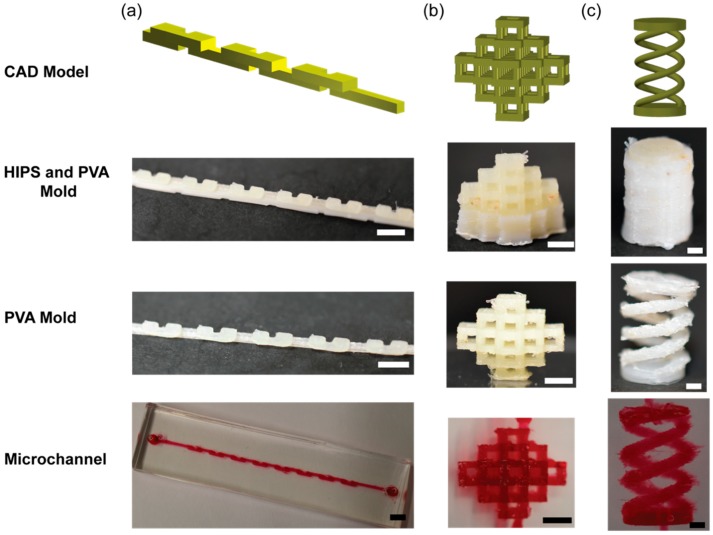
The CAD model of the three representative designs of the 3D microchannels, and optical images of the three major stages of dual sacrificial molding: after dual extrusion of HIPS (SM1) and PVA (SM2), after the removal of HIPS to obtain the PVA mold, and after the removal of PVA mold embedded in PDMS to obtain microchannels. (**a**) Serpentine mixer microchannel; (**b**) Multilayer pyramid network of microchannels; (**c**) Dual helix microchannel. The width and height of the channels with rectangular cross sections, denoted as (*w*, *h*), were (**a**) (600 μm, 800 μm) and (**b**) (800 μm, 800 μm). For (**c**), The diameter of the microchannel with circular cross sections was 800 μm. The dual helix mold was designed with 2.5 turns, a base radius of 5 mm and height of 20 mm. Scale bars = 3.0 mm for (**a**), 2.5 mm for (**b**), and 2.0 mm for (**c**).

We previously demonstrated that a single sacrificial mold made with PVA permitted to create 3D microchannels by preassembling modular pieces of sacrificial mold, and by including intricate designs directly onto the sacrificial mold [10]. PVA sacrificial molding has not yet demonstrated the fabrication of microchannels with complex 3D geometries with suspended features because of the lack of a method to pattern PVA in arbitrary 3D structures. Specifically, microchannels with an overhang or helical features are challenging to fabricate owing to the suspension of the structure in space, resulting in the need to incorporate complementary structures to support the components that are suspended in space. We demonstrated that dual sacrificial molding of HIPS and PVA can overcome this challenge. We fabricated selected geometries of microchannels that could not be fabricated using a single sacrificial material: serpentine, multilayer pyramidal network and dual helix. The overhang features of the serpentine channels (Figure 5a), the multilayer pyramidal network (Figure 5b) and the dual helical microchannels (Figure 5c) were supported by HIPS. After the removal of HIPS in limonene and PVA in water, an aqueous solution containing red dye was perfused to reveal microchannels (Figure 5a–c).

The duration required to remove HIPS and PVA sacrificial materials was dependent on the feature size presented in the sacrificial mold. In our work, the removal of HIPS from the dual sacrificial molds took 35 min for the serpentine channel, 24 h for the multilayer pyramidal network, and 24 h for the dual helix geometry. The removal of PVA in the PDMS matrix took 12 h for the serpentine channel, 36 h for the multilayer pyramidal network, and 36 h for the dual helix geometry. Overall, the duration required to complete the fabrication of the dual sacrificial mold and the subsequent evacuation of the mold in limonene and water to create microchannel largely relied on the design and dimension of the mold.

These demonstrations highlight the capability of dual sacrificial molding, which is not limited to specific geometries of the mold. The method presented here should provide an avenue to fabricate microchannels consisting of other free-hanging and arbitrary geometries that would not be attainable by a single step of sacrificial molding. In addition, the capability to remove PVA sacrificial mold solely by water widened the possibility of creating microchannels in other heat and photocurable polymer matrices and hydrogels [10].

## 5. Conclusions

In this paper, we proposed the concept of dual sacrificial molding and demonstrated the fabrication of microchannels with overhang and helical features by the proposed method. We used a FDM 3D printer to print the dual sacrificial mold using HIPS as the first sacrificial material (SM1) and PVA as the second sacrificial material (SM2) with the design of microchannels. The dual sacrificial mold was removed by dissolving HIPS in limonene initially to reveal the PVA sacrificial mold. We confirmed that the exposure of dual sacrificial mold, consisting of PVA and HIPS, to limonene had no adverse effects on the physical features patterned by PVA. The PVA sacrificial mold patterned with microchannels was embedded in PDMS and subsequently removed in water to reveal microchannels.

We designed dual sacrificial molding to obtain the final molds that were removable in water. One of the major advantages of this work is that the removal of the PVA sacrificial mold can be conveniently executed in aqueous conditions. As we demonstrated in the previous study [10], PVA sacrificial molding is shown to be useful to create microchannels in a plethora of matrix materials ranging from structural polymers to hydrogels. PVA is an excellent material as a sacrificial mold that can be dissolved in biologically relevant matrices such as cell-laden hydrogels. The method we developed here enabled to create microchannels with complex 3D geometries that were not attainable with simple PVA molds. As commercially available FDM 3D printers and filaments become low-cost and readily available, we envision that the method presented here will serve as an essential method to facilitate complex 3D biofabrication—fabrication of vascularized structures in biologically relevant and cell-laden matrices that serve as a platform from fundamental studies on cellular biology to applications in drug screening and discovery.
